# Tuning Intermediate Band Solar Cell Efficiency: The Interplay of Electric Fields, Composition, Impurities, and Confinement

**DOI:** 10.3390/nano14221858

**Published:** 2024-11-20

**Authors:** Hassan Abboudi, Redouane En-nadir, Mohamed A. Basyooni-M. Kabatas, Ayoub El Baraka, Ilyass Ez-zejjari, Haddou El Ghazi, Ahmed Sali

**Affiliations:** 1 Laboratory of Physic of Solids, Faculty of Science, Dhar El Mehrez University, Fes 30050, Morocco; 2 National School of Arts and Crafts Laboratory, National School of Arts and Crafts, Hassan II University, Casablanca 20360, Morocco; 3 Department of Precision and Microsystems Engineering, Delft University of Technology, Mekelweg 2, 2628 CD Delft, The Netherlands

**Keywords:** nitrides, IBSCs, efficiency, electric field, impurity, confinement

## Abstract

In this study, we investigated the influence of structural parameters, including active region dimensions, electric field intensity, In-composition, impurity position, and potential profiles, on the energy levels, sub-gap transitions, and photovoltaic characteristics of a p-GaN/i-(In, Ga)N/GaN-n (p-QW-n) structure. The finite element method (FEM) has been used to solve numerically the Schrödinger equation. We found that particle and sub-gap energy levels are susceptible to well width, electric field, and impurity position. Particle energy decreases with increasing well size and electric field intensity, while impurity position affects energy based on proximity to the well center. Potential profile shapes, such as rectangular (RQW) and parabolic (PQW), also play a significant role, with PQW profiles providing stronger particle confinement. IB width increases with electric field intensity and saturates at higher In-content. Voc increases with field strength but decreases with In-content, and the parabolic profile yields higher efficiency than the rectangular one. Photovoltaic efficiency is improved with an appropriately oriented electric field and decreases with higher In-content and field intensity. These findings highlight the critical role of structural parameters in optimizing QW-IBSC performance.

## 1. Introduction

One of the most existing renewable energy projects is photovoltaic because it is the most promising technology that directly converts solar radiation into electric energy. Indeed, solar energy is non-polluting and abundant enough that the amount of energy emitted by the Sun for one hour on the Earth’s surface is enough for the world’s energy requirements for one year. However, the current solar energy production does not exceed 1% of the global energy consumed. As a result, one major challenge for this technology is improving efficiency to exceed the Shockley–Quisser limit while maintaining a low production cost [1,2]. Third-generation solar cell technology based on different approaches, such as hot carriers, intermediate band, and multi-junction cells, has recently targeted the thermodynamic SC efficiency to reach or exceed the 86.6% limit. To improve the SC efficiency, minimizing different mechanisms of intrinsic losses, namely the losses below the band gap, thermalization, and emission, is necessary. From this perspective, many works are performed and reported in the literature [3,4,5,6,7]. Luque and Marti proposed in 1997 the intermediate band solar cells (IBSC) model as a fascinating way to improve the overall efficiency with an ideal photovoltaic conversion efficiency of 63.2% [8]. This theoretical success is attributed to using additional photons with energies below the band gap, which are typically considered unusable. In the last few decades, the advancements in nanotechnology, particularly the mastery of nanomaterial growth processes, have allowed the implant of quantum wells (QWs), quantum well wires (QWWs), and quantum dots (QDs) in the intrinsic area of p-i-n SC [7,9,10]. This enhances the photocurrent density and extends the optical absorption range without degrading the open circuit voltage [4,11]. In addition to quantum well wires of triangular cross-section, other promising systems for high-efficiency intermediate-band photovoltaic cells include dilute nitride III–V nanowire structures. These nanowires can potentially enhance performance by addressing thermalization losses and optimizing hot carrier transport, making them a valuable area for further exploration in the context of intermediate-band solar cells [12,13].

In this context, the nitride materials have recently attracted the attention of many researchers. Indeed, the (In, Ga)N material system exhibits excellent properties for small-dimension semiconductor devices. Also, it offers substantial potential in developing very high-efficiency solar cells for terrestrial and space applications due to its promising properties, such as adjustable bandgap, high resistance to radiation, great carriers’ mobility, and high absorption coefficient [14]. Deng et al. theoretically studied the photovoltaic conversion of (In,Ga)N/GaN QD SC system [15]. They showed that the optimum conversion efficiency is obtained with an intermediate band in the middle of the potential well by adjusting the QD size, inter-dot distance, and gallium content. Significant advancements have been made in modeling ternary IIIA nitrides in recent years, particularly concerning their electronic and optical properties. Notably, Filho et al. [16] explored the self-induced formation of core–shell InAlN nanorods using a combined density functional theory (DFT) and phase-field modeling (PFM) approach. Their work highlights the importance of structural, bonding, and electronic features at the nanometer scale. It demonstrates how DFT-derived parameters can effectively compute interfacial energies and diffusion coefficients essential for simulating nanostructured semiconductor systems. The insights gained from their research regarding phase separation, core/shell interfaces, and morphology provide a valuable framework for our study. While we focus on (In,Ga)N quantum wells as intermediate band solar cells (IBSCs), the principles established by Filho et al. [17] regarding composition and confinement effects reinforce the relevance of our findings. Further, Chowdhury et al. have proposed an InGaN/GaN IBSC via the Kroning–Penny model-based resolution of the Schrodinger equation to obtain high efficiency [8]. They have found that the maximum conversion efficiency reached approximately 63.14%. Moreover, using an InAs/GaAs doping system, Imran et al. have considered the intermediate band’s flatness control and revealed a maximum efficiency of 44.92% [9]. In addition, Miller et al. have investigated the electric field perpendicular and parallel to the layer dependence on optical absorption in the band gap of quantum well structures [18]. More recently, El Ghazi et al. studied the performance of GaN/InN/GaN-based IBSC in the presence of the electric field and impurity. The modeling study revealed maximum photovoltaic conversion efficiency for a thin QW, a large barrier, and an intense field [19].

This study introduces a novel approach to optimizing the performance of (In,Ga)N/GaN quantum well-based intermediate band solar cells (IBSC) by explicitly examining the critical influence of electric field intensity, heavy holes, and impurities—factors frequently overlooked in existing literature. Unlike previous research, our investigation delves into how these elements interact with indium concentration and structural parameters and the type and form of confinement potential energy. By highlighting these interactions, we aim to provide a more comprehensive understanding of their effects on the efficiency and functionality of IBSC, thus advancing the development of next-generation solar cell technologies. This article is structured as follows: Section 2 presents the theoretical framework, Section 3 presents the results and discussion, and finally, Section 4 provides the concluding remarks.

## 2. Theoretical Background

**Figure 1:**
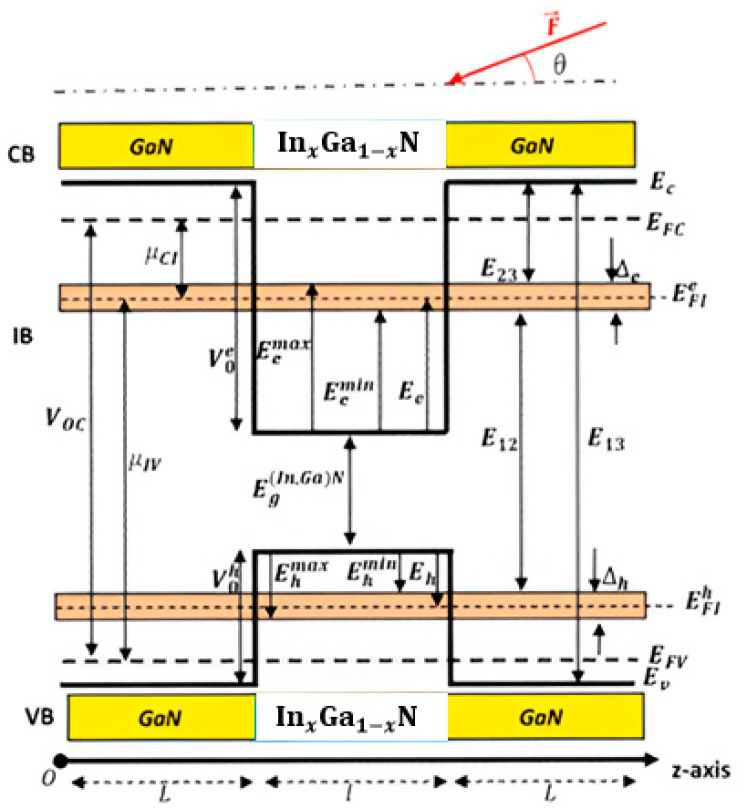
Schematic diagram of GaN/InGaN/GaN QW one-intermediate band integrated into the conventional solar cell under study considering the quantum well width l, barriers height ${\;V}_{o}^{e,h}$, and built-in electric field $\vec{F}$ (θ=0°).

Our reference system is a p-GaN/(In,Ga)N/GaN-n solar cell illustrated in Figure 1. Such a structure is characterized by a GaN type layer with a thickness L (barrier with) and an intrinsic (In,Ga)N type layer with a thickness l (well width). Without losing generality, the influences of n(p)-type doping via the Poisson equation are neglected in this paper. This structure constitutes a quantum-well intermediate-band solar cell (QW-IBSC) model, as depicted in Figure 1. It allows the absorption of three photons via two steps, leading to much higher absorption than a conventional p-n structure. As shown in Figure 1, $\;E_{C}\;,E_{v}\;,\;E_{Fc},\;and\;E_{Fv}$ represent, respectively, the energies of the conduction band, valence band, and their quasi-Fermi levels, while $E_{FI}$ represents the IB quasi-Fermi level energy. CB and VB denote the conduction and valence bands, respectively. ${µ}_{CI}$ and ${µ}_{IV}$ are chemical potentials between the CB and the IB, respectively, and $\mu_{CV}$ is the chemical potential between the CB and VB. $E_{13},\;E_{23},$ and $E_{12}$ represent the bottom and top sub-bandgap energy, respectively.

The energy levels and their corresponding eigenfunctions in such a system are determined by solving the time-independent Schrodinger equation:

(1) Hψ=Eψ

Within the parabolic band and the effective mass approximations, the Hamiltonian of a single particle in the ${In}_{x}{Ga}_{1-x}N$/GaN QW structure under the impurity and the electric field effects concerning the indium content (x) and temperature (T) impacts is written as follows:

(2) $$H_{i}=-\frac{\hbar^{2}}{2}\vec{\nabla }\left(\frac{1}{2m_{i}^{*}\left(x\right)}\vec{\nabla }\right)+V_{0}^{i}\pm \frac{e^{2}}{\varepsilon^{*}{\left(x\right)\varepsilon }_{0}\left|{\vec{r}}_{i}-{\vec{r}}_{0}\right|}\pm \;e\;F\;r_{i}\cos\left(\theta \right)\left(i\equiv e,h\right)$$

where $\vec{F\;}$ is the built-in electric field’s intensity; θ is the angle between $\vec{F}$ and ${\vec{r}}_{i}$; e is the absolute electron charge; and ${\vec{r}}_{0}\left(x_{0},y_{0},z_{0}\right)$ and ${\vec{r}}_{i}\left(x_{i},y_{i},z_{i}\right)$ are the impurity and particle position, respectively. $m_{i}^{*}$ is the effective mass; $\varepsilon_{0}$ is the vacuum permittivity; $\varepsilon^{*}$ is the relative dielectric constant; and $V_{0}^{i}$ is the particle-related potential confinement.

We have adopted a dimensionless parameter considering the electric field effect to simplify. $\left(\mu =\frac{ea^{*}F}{R_{b}^{*}}\right)$ and the Bohr radius $\left(a_{b}^{*}=\frac{{4\pi \varepsilon }_{0}\hbar^{2}}{m^{*}{\cdot \;e}^{2}}\approx 2.57\;nm\right)$ as the unit of size, and the effective Rydberg $\left(R_{b}^{*}=\frac{m_{b}^{*}e^{4}}{2(4\pi \varepsilon_{b}^{*}{\hbar )}^{2}}\approx 29\;meV\right)$ is used as unit energy. $m_{i}^{*}\left(x\right)$ and $\varepsilon^{*}\left(x\right)$ are, respectively, the temperature- and indium-content-dependent effective masses and main dielectric constants. The ternary (In,Ga)N is the linear combination of InN and GaN materials.

In this study, we have also considered two different potential profiles. Parabolic quantum well (parab. QW or PQW) and rectangular quantum well (rect. QW or RQW). Their analytical expressions are given, respectively, as follows:

(3) $$V_{0P}^{i}\left(z\right)=\left\{\begin{array}{c} \frac{4Q^{i}∆E_{g}\left(x\right)}{l^{2}}{\left(z_{i}-L-\frac{l}{2}\right)}^{2}for\;\;\;\;L\;\leq z_{i}\leq L+l\;\\ Q^{i}∆E_{g}\left(x\right)\;\;\;\;\;\;\;\;\;\;\;\;\;\;\;\;\;\;\;\;\;\;\;\;\;\;\;\;\;\;\;\;\;\;\;\;\;\;\;\;\;elsewhere\; \end{array}\right.$$

(4) $$V_{0R}^{i}\left(z\right)=\left\{\begin{array}{c} 0\;\;\;\;\;\;\;\;\;\;\;\;\;\;\;\;\;\;\;\;\;\;\;\;\;\;\;\;\;\;\;\;\;\;\;\;\;\;for\;\;\;\;\;\;L\;\leq z\leq L+l\;\\ Q^{i}∆E_{g}\left(x\right)\;\;\;\;\;\;\;\;\;\;\;\;\;\;\;\;\;\;\;\;\;\;\;\;\;\;\;\;\;\;\;\;\;\;\;\;\;\;\;\;\;elsewhere\; \end{array}\right.$$

where $Q^{i}=0.7(0.3)$ is the conduction (valence) band offset, and $∆E_{g}\left(x,T\right)$ is the band-gap energy difference between GaN  and ${In}_{x}Ga_{1-x}N$, governed by the In-fraction and temperature given as [20]:

(5) $$∆E_{g}\left(x\right)=E_{g}^{GaN}\left(x\right)-E_{g}^{{In}_{x}Ga_{1-x}N}\left(x\right)$$

The ${In}_{x}Ga_{1-x}N$ band gap energy is expressed using the following quadratic function of the In-concentration as follows [21,22]:

(6) $$E_{g}^{{In}_{x}Ga_{1-x}N}\left(x,\right)=x\cdot E_{g}^{InN}+{\left(1-x\right)\cdot E}_{g}^{GaN}-Cx\left(1-x\right)$$

where $C\left(=1.43\;eV\right)$ is the band gap bowing parameter. $E_{g}^{InN}$ and $E_{g}^{GaN}$ are the band gap energies of InN and GaN, respectively. The effective masses of InGaN depend on the indium concentration, with the x-fraction of indium represented as follows:

(7) $$m_{InGaN}^{*}\left(x,\;T\right)=x\cdot m_{InN}^{*}\left(x\right)+\left(1-x\right)m_{GaN}^{*}\left(x\right)$$

The presence of impurity makes the Schrödinger equation unsolvable analytically. Thus, the finite element method (FEM) is devoted to solving this problem considering a mono-directional mesh (calculation grid) composed of 3N+1 points with N=200 points. It provides accurate results for the ground and low-lying excited states of simple quantum systems such as the harmonic oscillator or the particle in a box. However, for more complex systems or higher excited states, the accuracy of the FEM solution may decrease due to the need for a finer mesh and higher-order basis functions. Overall, the accuracy of this method depends on the problem’s complexity, the choice of numerical parameters, and the computational resources available. Notice that to obtain the energy levels and their corresponding wave functions, the  z−axis 1D-Schrödinger equation is numerically solved considering the following boundary conditions:

(8) $${\left[\underset{n}{\to }.\underset{\nabla }{\to }\left(\frac{\psi }{m_{e,b}^{*}}\right)\right]}_{b}={\left[\underset{n}{\to }.\underset{\nabla }{\to }\left(\frac{\psi }{m_{e,w}^{*}}\right)\right]}_{w}$$

The mesh grid of 3N+1 points is considered for the studied system. Various discretization steps discretize each layer. For the barriers, the step is $h_{b}=L/N$, while for the well’s regions, it is given as $h_{w}=l/N$. Therefore, for 0<k<N, the mesh’s nodes of single QW are given, respectively, as follows: the left barrier is $z_{k}=k\;*\;h_{b}$, in the well region is $z_{k}=L+k\;*\;h_{w}$, and in the right barrier is given as $z_{k}=L+l+k\;*\;h_{b}$. The first and second derivative wave functions are calculated using the finite element method [23]:

(9) $${\left(\frac{\partial 2\psi (z)}{\partial z2}\right)}_{z_{k}}=\frac{\psi_{k+1}-2\psi_{k}+\psi_{k-1}}{{\left(z_{k+1}-z_{k}\right)}^{2}}$$

(10) $${\left(\frac{\partial \psi \left(z\right)}{\partial z}\right)}_{z_{k}}=\frac{\psi_{k+1}-\psi_{k}}{z_{k+1}-z_{k}}$$

A large absorption of the light spectrum is required to optimize photoelectric conversion efficiency. Indeed, three distinct optical transitions appear in such a structure: VB to IB, IB to CB, and VB to CB. For the standard SC, only photons with energy higher than the band gap energy can generate electron–hole pairs. However, for the IBSC system, the photons with energies less than the band gap energy can be absorbed via the first and second optical transitions [24]. In light of what has been described above and for proper operation of IBSC. Certain assumptions must be maintained, such as the IB strip being electrically isolated from external contacts and no current being extracted from the IB. All transitions between VB, IB, and CB must be radiative, the quasi-Fermi levels corresponding to each band must be constant, and the device must be thick enough to ensure total absorption. Indeed, the photocurrent density, open circuit voltage, and efficiency are the most exciting parameters of solar cells. Furthermore, under full-concentration sunlight, the number of absorbed and emitted photons determines the photo-generated density by the solar cell. Referring to the IBSC energy band diagram described in Figure 1, $\;j_{sc}$ can be expressed as follows [25]:

(11) $$\frac{j_{sc}}{e}=\left[N\left(E_{13},\infty ,T_{s},0\right)-N\left(E_{13},\infty ,T_{c},\mu_{CV}\right)\right]+\left[N\left(E_{23},E_{12},T_{s},0\right)-N\left(E_{23},E_{12},T_{c},\mu_{CI}\right)\right]$$

where $T_{S}$ and $T_{C}$ are, respectively, the surface temperatures of the Sun and the solar cell; $\mu_{CV}$ is the chemical potential between the CB and VB; and $\mu_{CI}$ is the chemical potential between the IB and CB. Notice that all transition energies ($E_{13}$, $E_{12}$, and $E_{23}$) are specified in Figure 1. Moreover, according to the Roosbroeck–Shockley formula, the flux N of photons leaving an object at temperature T is expressed as [2]:

(12) $$N\left(E_{a},E_{b},T,\mu \right)=\frac{2\pi }{h^{3}c^{2}}\int_{E_{a}}^{E_{b}}\frac{E^{2}dE}{e^{\frac{\left(E-\mu \right)}{k_{B}T}}-1}$$

where $E_{a}$ and $E_{b}$ are, respectively, the lower and upper energy limits of the photon flux for the corresponding transitions ; T is the temperature; h is the Plank’s constant; c is the light speed in vacuum; $k_{B}\;(\approx 1.38\;\times \;10^{-23}\;J/K)$ is the Boltzmann’s constant; and μ is the chemical potential of the transition.

On the other hand, the IBSC output voltage $\left(V_{oc}\right)$ of a p−i−n solar cell can be written as follows [26]:

(13) $$V_{oc}=\mu_{CV}=\mu_{CI}+{\mu \;}_{IV}$$

where $\mu_{CI}$ and $\mu_{IV}$ are given as follows:

(14) $$\left\{\begin{array}{c} \mu_{CI}=E_{23}+0.5∆e-E_{c}+E_{FC}\\ \mu_{IV}=E_{12}+0.5∆e-E_{FV}+E_{V}+V_{0h}+E_{h}^{1} \end{array}\right.$$

The election IB width is noted as ∆e.

The quasi-Fermi levels $E_{FC}$ and $E_{FV}$ of the CB and the VB, respectively, can be expressed as follows [27]:

(15) $$\left\{\begin{array}{c} E_{c}-E_{Fc}=kT\ln\left(\frac{N_{c}}{n}\right)\\ {\;E}_{FV}-E_{V}=kT\ln\left(\frac{N_{V}}{p}\right) \end{array}\right.$$

where $N_{c}$ and $N_{V}$ are the effective CB and VB densities, respectively, while n and p are, respectively, the electron and hole concentrations given as follows [28]:

(16) $$\left\{\begin{array}{c} n=N_{c}\;exp\left[-\frac{Q∆E_{g}\left(x\right)}{K_{B}T}\right]\\ p=N_{V}\;exp\left[-\frac{(1-Q)∆E_{g}\left(x\right)}{K_{B}T}\right] \end{array}\right.$$

Also, the CB and VB effective densities are expressed as follows:

(17) Nc=Nc* T32NV=NV* T32

To be more realistic, mainly in previous studies related to the theoretical efficiency of solar cells, the fill factor was often assumed to be 1, which means that the entire surface of the solar cell was considered to be active and capable of converting sunlight into electric energy. However, in reality, the fill factor is always less than 1 because there are always inactive areas on the cell surface. However, we have used the following fill factor (FF) expression depending on the open-circuit voltage with $V_{th}=\frac{K_{B}T}{q}$ [29]:

(18) $$FF=\frac{\frac{V_{oc}}{V_{th}}-ln\left(\frac{V_{oc}}{V_{th}}+0.72\right)}{\;1+\frac{V_{oc}}{V_{th}}}$$

Therefore, the photovoltaic conversion efficiency can be obtained using the output voltage and photo-current density as follows [30]:

(19) $$\eta =\frac{V_{oc}\cdot J_{sc}\cdot FF}{P_{in}}$$

We notice that $P_{in}$ is the incident of Sun power per area unit given by Stefan–Boltzman’s law $P_{in}=\sigma T_{s}^{4},$ where $\sigma =5.67\cdot 10^{-8}\;Wm^{-2}K^{-4}$.

## 3. Results and Discussion

**Figure 2:**
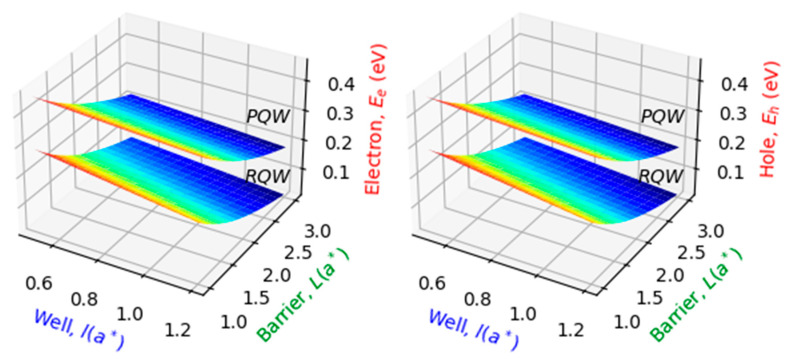
The ground-state energy for electron (**left**) and heavy hole (**right**) versus the well and barrier widths for two potential profiles (PQW, RQW) with an on-center impurity and T=300 K, x=20% and μ=0.5 for θ=0°.

The reference range of p-GaN/i-(In, Ga)N/GaN-n (p-QW-n) structure parameters used to validate and investigate numerically our theoretical modeling is cited in Table 1. Our results deal principally with the electric field, In-content, and impurity’s position dependent on the particle’s (electron, hole) energy level, bottom and top sub-gap energies, and CB longitudinal profile. Concerning the photovoltaic results, they are mainly concerned with the IB width, open circuit voltage, current density, and photovoltaic conversion efficiency. Based on the proposed model, it is evident that the electron (hole) energy constitutes the important key to computing the IBSC performances. In this trend, let us start with the results regarding the particle energy levels obtained via these parameters. Panels of Figure 2 illustrate, respectively, the variations of the electron and hole ground state energies for In-content (x=0.2) and electric field (μ=0.5) at room temperature versus structure size. The influence of the shape is also considered via the rectangular and parabolic profiles. Regardless of the structure size, it can be seen that the particle energy in PQW is more significant than that in RQW, as expected. The physical reason can be due to the strong confinement in a parabolic shape compared to a rectangular one. Furthermore, it is observed from both panels that the particle energy decreases with the increase in structure size, regardless of shape.

**Figure 3:**
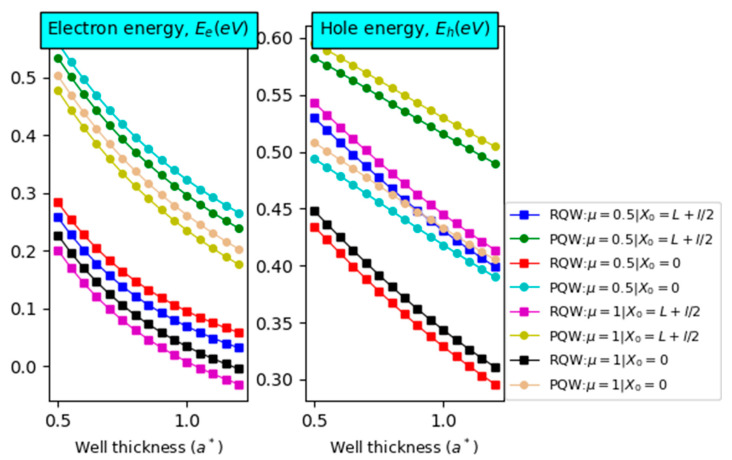
The energy of the electron (**left panel**) and hole (**right panel**) versus the well width in GaN/${In}_{0.4}{Ga}_{0.6}$N/GaN QW−IBSC for two potential profiles. Two values of impurity’s positions and electric field are considered with *L* = 3$a^{*}$,  x=40%, and T = 300 K for θ=0°.

To calculate the IBSC characteristics under different perturbations, we primarily display the particle energy versus the well size for two different values of the electric field: impurity position and profile. The calculations are performed at room temperature for In-content x=0.40 and a barrier width of $L=3a^{*}$. As expected, both panels of Figure 3 show a remarkable drop in the particle energy with increasing well width regardless of the electric field intensity, impurity’s position, and confinement profile. For all values of $X_{0}$, it appears that $E_{e}\left(E_{h}\right)$ decreases (increases) with increasing the electric field $\left(\mu \right)$. Moreover, it is observed that particle eigenvalue is strongly impurity position-dependent. Indeed, as the impurity is moved far from the well-center, $E_{e}\left(E_{h}\right)$ decreases (increases).

**Figure 4:**
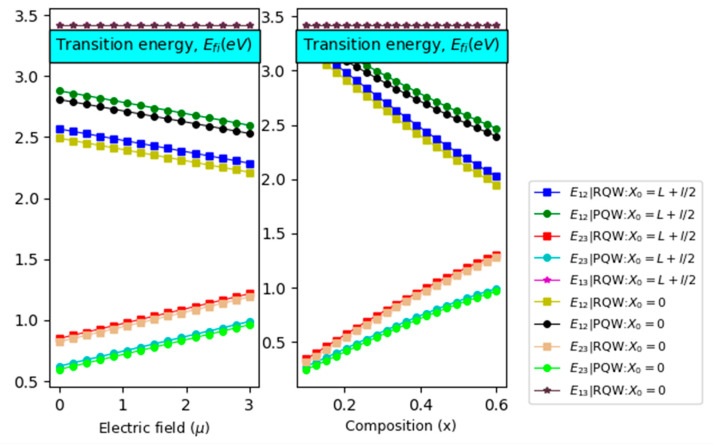
Transition energy (sub-gaps) versus the electric field (**left panel**) with x=40%  and compositions (**right panel**) with µ = 1 considering the effects of impurity position for two potential profiles (RQW and PQW) with $L=3l=3a^{*}\;$ for θ=0°.

After presenting the particle energy results necessary to calculate different IBSC characteristics, let us discuss different optical transitions. $\left(E_{12},\;E_{23},\;and\;E_{13}\right)$ as displayed in Figure 4. The right and left panels illustrate their variations according to the electric field and compositions. The obtained results are carried out for two different potential profiles and two impurity positions with $\;L=3\times l=3a^{*}$ and T=300 K. As expected, $E_{13}$ remains intact under the variation of internal and external excitations, especially for dimensionless electric fields less than  µ=3. Furthermore, it is clearly shown from the left panel that $E_{12}$ is linearly reduced with increasing the electric field intensity, despite the consequences of the shape and impurity position. This outcome can be understood based on the particle energy discussed above (Figure 3). Furthermore, we notice that for a parabolic profile and an impurity located at the band edge of the left barrier, $E_{12}$ achieves its highest value. Compared to the significant potential profile impact, impurity has consequential influences.

**Figure 5:**
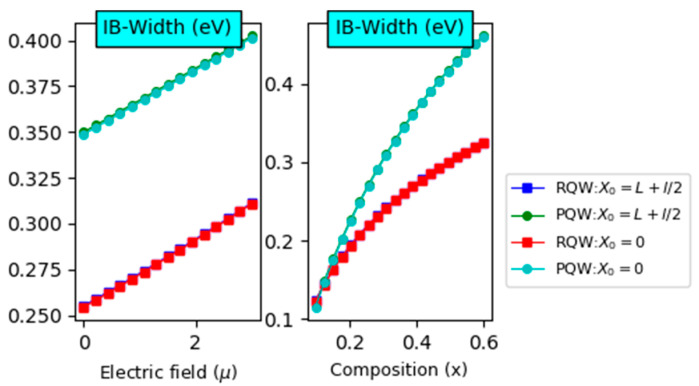
Intermediate band (IB width) as a function of the electric field (**left panel**) with x=40% and compositions (**right panel**) with µ = 1 for two shapes (RQW and PQW). Two impurities (s positions are considered with $\;L=3\times l=3a^{*}\;$ for θ=0°.

However, the optimized value of $E_{23}$ is obtained for a rectangular potential regardless of the impurity position. On the other hand, the right panel shows that $E_{12}$($E_{23}$) is found to be reduced (improved) linearly with increasing the In-content. Such behavior can be assigned to the particle energy and the confinement potential height versus the In-content. It is interesting to notice that $E_{12}$ is more marked for on-center impurities compared to off-center ones. In addition, it should be emphasized that the potential profile impact surpasses the effect of the impurity’s position. Indeed, the parabolic profile and the on-center impurity yield the best results. For ${\;E}_{23}$, it appears that the greatest value is attained for a rectangular profile with negligible effect on the impurity’s position. The left and right panels of Figure 5 illustrate the variation of IB width versus the electric field intensity and the chemical composition, respectively. The calculations have been performed for two different impurity positions and rectangular and parabolic profiles. It is observed that the IB width increases linearly with increasing the electric field intensity, while it tends to saturation regime for high chemical content.

**Figure 6:**
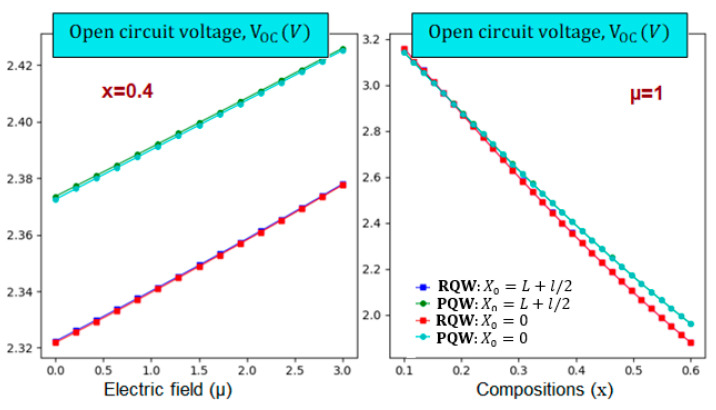
The open circuit voltage variation versus the electric field intensity (**left**) and In-content (**right**) for two different impurity positions and two different profiles. $T=300\;K,\;and\;L=3l=3a^{*}$ for θ=0°.

**Figure 7:**
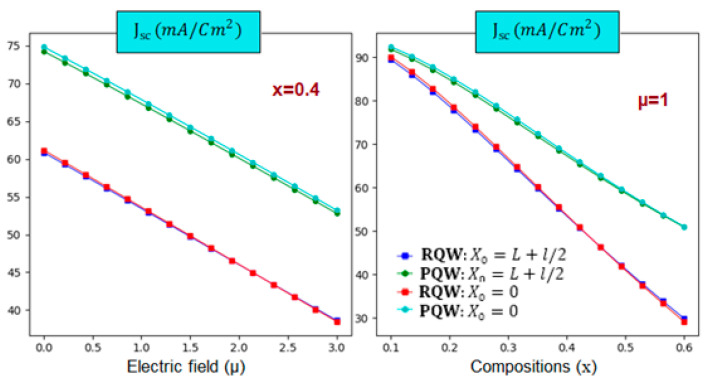
The variation of the short-circuit current density as a function of electric field strength (**left**) and In-content (**right**) for two different impurity positions considering the shape effect at room temperature. $T=300\;K,\;and\;L=3l=3a^{*}$ for θ=0°.

Furthermore, we notice that the profile impact is more pronounced than the displacement of impurity positions. Additionally, the parabolic-related IB is wider for fixed electric field strength than the rectangular one. On the other hand, the right panel reveals a crossover point around 0.18, limiting to different behaviors. For In-content that is less than the critical value, the parabolic-related IB width is greater than the rectangular one. However, this behavior is inverted for high In-content. After discussing the main keys necessary to perform the calculations of the solar cell parameters, let us now start with the short circuit current density ($J_{sc}$) and the open circuit voltage ($V_{oc}$). Indeed, panels of Figure 6 display the $V_{oc}$ variations according to the electric field and In-content at room temperature for two profiles. It should be noted that it is linearly enhanced (dropped) versus the electric field (In-content) despite the consequences of the profile and impurity’s position. In addition, the right panel illustrates that it is the largest for parabolic profile and high electric field value. Moreover, the left panel shows that the In-content drop rate is more noteworthy than the electric field enhancement rate. Figure 7 displays the variations of the photo-generated current density $\left(j_{sc}\right)$ According to the electric field and In-composition for the rectangular and parabolic profiles at room temperature and L=3l=3. The main characteristic is that. $j_{sc}$ decreases with increasing these parameters apart from the profile and impurity position. In addition, it is obvious that $j_{sc}$ is more significant for the parabolic profile. 

For instance, with increasing µ from zero to 3, it is found that $j_{sc}$ drops from 74.5 (60.92) to $53.14\;\left(38.66\right)\;mA\cdot {cm}^{-2}$ shown a noticeable drop of about $28.7\%\;\left(36.5\%\right)$ for the parabolic (rectangular) profile. In the same trend, $j_{sc}$ is found to be reduced from 90 (92) to 28 (52) $mA\cdot {cm}^{-2}$ with increasing the In-composition from 10% to 60%, indicating a significant reduction of about $69\%\;\left(43\%\right)$ for rectangular (parabolic) shape. It is seen that the rectangular shape is more In-content dependent than the parabolic one. However, the same drop tendency versus the electric field is shown regardless of the shape and impurity position.

**Figure 8:**
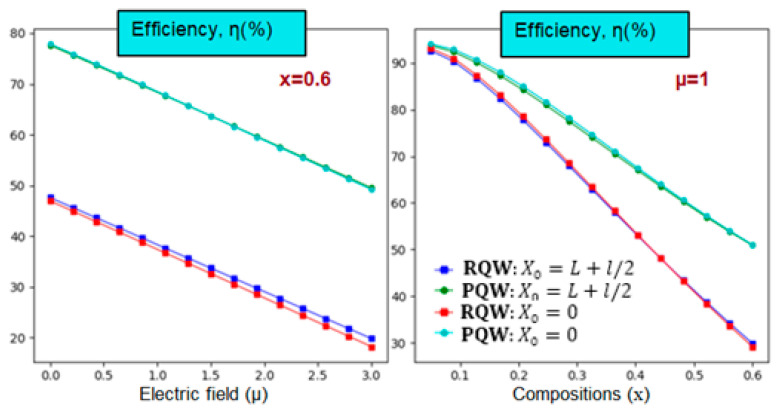
Photovoltaic conversion efficiency versus the electric field (**left**) and In-content (**right**) at room temperature considering two different impurity positions and two types of potentials. $L=3l=3a^{*}$ for θ=0°.

Now, we turn to the most crucial variable for QW-IBSC after examining and discussing open-circuit voltage and photo-generated current density. The numerical findings for photovoltaic conversion efficiency versus In-content (right) and electric field (left) are illustrated in Figure 8. Calculations are carried out at room temperature with a structure size of L=3l=3. It is obvious that the efficiency curves in Figure 8 are heavily influenced by those of $V_{co}$ and $J_{sc}$. Furthermore, it is observed that the optimum efficiency value is obtained for parabolic potential. Additionally, increasing the In-content makes the rectangular-related efficiency drop rate more significant for rectangular shapes than parabolic ones. Accordingly, it is noticeable, regardless of the other parameters, that the maximum photovoltaic efficiency is obtained without applying electric field intensity (µ=0).

For instance, when the electric field intensity is increased from µ=0 to µ=3, the efficiency drops by 36.27%, from 77.57 to 49.43 for parabolic potential. In fact, with an impurity placed at the left barrier edge (on-center), the efficiency decreases from $47.87\left(47.17\right)$ to $19.90\left(17.99\right)$ showing a reduction of $58.43\left(61.86\right)\%$ for the rectangular profile. The efficiency fluctuations are then depicted in the left panel according to the In-content for a given electric field intensity, considering the impurity’s position and confinement potential profile influences. As the In-content rises, efficiency diminishes regardless of the impurity position. For instance, as the In-content increases from 10 to 60%, efficiency decreases from 91.99 $\left(89.78\right)$ to 50.42 $\left(29.08\right)$, showing an essential reduction of $45.19\left(67.61\right)\%$ for the parabolic (rectangular) profile. These results can be understood based on the principle of inter-band transition shrink, which has moved toward lower energies, affecting the position and width of the IB and, hence, the performance of our solar cells.

**Figure 9:**
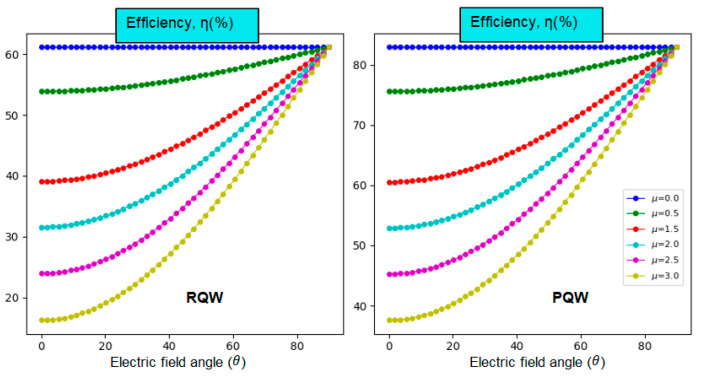
On-center impurity-related photovoltaic conversion efficiency versus the angle θ for different values of electric field strength and two different forms of potentials at room temperature. $x=0.5,\;and\;L=3l=3a^{*}$.

The orientation of the built-in electric field along the growth direction, specifically the *z*-axis component, is known to significantly influence the optical and electrical properties of nanostructures, particularly quantum wells. In Figure 9, we illustrate the impact of the electric field angle on the solar cell’s performance while keeping all other parameters constant. Both profiles exhibit a similar trend in efficiency as a function of the electric field angle. The efficiency remains unchanged for µ=0 (θ=π⁄2). It nonlinearly increases as the angle is increased, eventually converging to the same value at θ=π⁄2, which corresponds to the maximum efficiency of the solar cell [35]. This is expected due to the absence of Stark-effect confinement. Notice that the angle impact is more manifested for small angles than greater ones.

Overall, a change in $V_{oc},\;\;J_{sc},$ and FF under electric field orientation all contribute to tuning the power output and, ultimately, the efficiency of the solar cell. Therefore, optimizing these physical quantities can lead to more efficient solar cells. We may categorically state without losing generality that our results are relatively superior to the real returns since our calculations are assumed to be performed under ideal conditions. Additionally, we must remember that radiative recombination and manufacturing defects (stresses), not discussed above, can all impact solar cell performance.

## 4. Conclusions

Our study reveals the critical influence of key structural parameters on the energy levels, sub-gap transitions, and photovoltaic performance of the p-GaN/i-(In, Ga)N/GaN-n (p-QW-n) structure. Moreover, our study reveals the following: (i) Particle energy levels are susceptible to well width, electric field intensity, and impurity position. Larger well sizes and stronger electric fields lead to lower energy levels. (ii) Parabolic potential profiles (PQW) provide enhanced particle confinement and higher energy levels compared to rectangular profiles (RQW), which significantly improve performance. (iii) The intermediate band (IB) width increases with electric field intensity, saturating at higher In-content, indicating effective electric field tuning for optimizing IB properties. (iv) Higher photovoltaic efficiency is achieved with parabolic profiles, peaking without an oriented electric field and diminishing with increased In-content and electric field strength. These findings show the importance of structural parameters and applied electromagnetic fields in optimizing the energy landscape, especially QW-IBSCs-based photovoltaic efficiency. Future research should investigate how band bending impacts carrier distribution and energy levels while analyzing the influence of impurity distributions on energy band alignment and transitions to improve our understanding of photovoltaic performance.

## Figures and Tables

**Table 1:** The main physical parameters used in our calculations [31,32,33,34].

Parameters	GaN	InN
Eg (eV)	3.42	0.78
$\varepsilon^{*}$	8.68	11.6
m*_e_/m_0_	0.19	0.05
m*_h_/m_0_	0.81	0.83
N^0^_v_ (m^−3^)	8 × 10^21^	10^22^
N^0^_c_ (m^−3^)	2.3 × 10^20^	1.76 × 10^20^

## Data Availability

Data are contained within the article.
